# Seeing Context through Metaphor: Using Communications Research to Bring a Social Determinants Perspective to Public Thinking about Child Abuse and Neglect

**DOI:** 10.3390/ijerph15010152

**Published:** 2018-01-19

**Authors:** Nathaniel Kendall-Taylor, Kate Stanley

**Affiliations:** 1 The FrameWorks Institute, 1333 H St NW, Washington, DC 20005, USA; 2 The National Society for the Prevention of Cruelty to Children, Weston House, 42 Curtain Road, London EC2A 3NH, UK; kate.stanley@nspcc.org.uk

**Keywords:** communication science, culture and health, social determinants, child abuse and neglect, metaphor, science translation, public policy

## Abstract

Human beings think in metaphor and reason through analogy. The metaphors through which we think influence how we understand and feel about social issues as well as the actions that we see as appropriate and important. Metaphors can be used to increase understanding of how issues work and increase the salience of a given issue, build support for programs and policies necessary to address the issue, and instigate demand for change and civic action. In this paper, we use a mixed methods research design, including brief qualitative interviews, experimental surveys, and focus groups, to test the ability of different metaphors to influence public understanding of the social determinants of child abuse and neglect in the UK. We find one metaphor in particular that improves people’s understanding of the social causes of child maltreatment and increases support for structural solutions. This metaphor can be used to build support for preventative public health solutions.

## 1. Introduction

The UK public widely supports the idea that child abuse and neglect is a prevalent and disturbing social problem about which something needs to be done [1,2]. However, the solutions that people identify as appropriate and potentially effective tend to focus narrowly at the family level: removing children from the home, providing parents with more information, or improving parenting skills. There is a general lack of recognition of child maltreatment as a public health issue shaped by social determinants. There is a corresponding lack of focus on the role and potential for these issues to be addressed via public programs and policy [1]. 

Our research explores the ability of metaphor to address this lack of a social determinants perspective. At its core, our work is about increasing the availability and applicability of information in order to foster a richer and more robust public discussion about what could be done to better address and prevent child abuse and neglect. Bringing a deeper appreciation of social determinants to public thinking and into the public discourse has applications and potential utility on other issues where social determinants play a causal and remedial role.

Despite the lack of public understanding of the role of social determinants, research has shown that child maltreatment is often associated with systemic influences such as poverty, health inequalities, and social isolation [3,4]. Likewise, it has also been suggested that addressing social determinants is an important part of a comprehensive public health strategy to prevent child maltreatment and reduce children’s exposure to the early adverse experiences that can derail development and negatively affect long-term health and wellbeing [5,6,7,8,9].

The relative absence of a social determinants perspective among non-expert audiences may be characteristic of thinking about public health issues more generally [10]. Research has shown that people tend to focus on the agency of individuals and personal responsibility rather than the structural, social, or cultural determinants of health [11,12]. This focus at the individual level and inattention to context likely limits public understanding of child maltreatment as a public health issue, one that is influenced by systemic, economic, and social factors and is amenable to change via the remediation of these factors [13,14,15].

Clearly and effectively communicating the role that contextual factors play in causing child abuse and neglect can be seen as a public health intervention strategy. An improved recognition of the social dimensions of the issue can open the possibility for policy and practice change that might more effectively prevent and address child abuse and neglect. Improving public understanding is thus not only important in changing individual behaviors, but also in increasing issue salience and building demand for structural change [16,17]. 

While important from a number of perspectives, using communications to improve understanding of the role of social conditions in child maltreatment is challenging. It is relatively easy for non-expert audiences to see how individual factors such as poor decision-making and lack of personal control can lead to acts of child maltreatment. It is decidedly more difficult to recognize larger social forces like poverty, health inequalities, and social isolation as contributing causes of this social issue or as locations for potential solutions [1,2,18].

For those working in the UK to improve the lives of children, families, and communities, promoting a better understanding of how social factors play a causal role in child maltreatment has become core work. Building a better understanding of how social factors influence this set of issues can help the public and decision makers see the importance of and get behind programs that target these causal factors. 

The research presented here is part of a larger strategy being pursued by the National Society for the Prevention of Cruelty to Children (NSPCC), a non-profit organization working to end child abuse and neglect in the UK. The organization has realized that it is not enough for people to be aware of child abuse and neglect. It is now focused on creating an understanding that the issue is addressable and is working to build wider support for the changes that are necessary to do so. To mobilize support for solutions that will help prevent child abuse and better protect children when acts of maltreatment do occur, members of the public need to be equipped with a better understanding of the causes, consequences, and possible solutions to child abuse. For example, when a person considers the primary cause of child abuse to human “evil”, there is little space to see the importance of and role for the policies and programs that research show to be effective. When people recognize the role of social factors that are at play in these issues—for example, the ways in which poor mental health heightens the risk of child abuse—a wider range of policy and practice solutions becomes visible and can be seen as appropriate and effective. 

## 2. Background 

As members of the public and policymakers consider social policies intended to address and prevent child abuse and neglect, the way in which these issues are framed becomes highly relevant [19,20,21]. Message framing—or the choices made in how information is presented and what is said and left unsaid—can play a powerful role in how people understand a target issue and the solutions they support [22]. Framing can improve issue understanding and serve as a corrective to patterns of thinking that prevent people from seeing the importance of context, systems and policies in solving social problems [23,24,25]. In expanding understandings of how issues work, issue framing can encourage productive consideration of social policy issues, drive healthy debate of policy approaches, and increase support and demand for necessary solutions [26]. Issue framing is thus an important tool in creating public support for evidence-based policies and programs, and in turn can play a role in reforming systems to better address public health problems. 

The use of metaphor is one common method of framing social issues [27]. In this capacity, metaphors re-categorize a target concept in terms of another concept, group of concepts or conceptual domain [28,29,30,31,32]. By using metaphor, communicators can help people think and talk productively about complex and unfamiliar issues in more proficient and informed ways [33,34]. In this applied function, metaphors tap into the features of a concrete, familiar thing or category of things and export the most salient features of these concepts to a second more abstract, complex or unfamiliar domain, thereby making it more familiar, understandable, and applicable [35]. Metaphors take groups of operational meanings and transfer them into a domain with which listeners or readers are unfamiliar, providing associations that can be used in understanding issues and communicating with others [36].

In the multi-method research process described below, we sought to generate a metaphor that could be used to increase public understanding of the social determinants of child abuse and neglect in the UK. The end goal of this research was to provide those communicating about children’s issues in the UK with a communications tool that could be used to increase support for policies and programs that can prevent child maltreatment and better address the negative effects of significant early adversity on children’s development and long-term outcomes. Our specific research question was: can metaphor be used to improve understanding of the social determinants of child maltreatment and increase support for preventative policies and practice? 

## 3. Research Methods and Findings

To answer this research question, we (1) developed candidate metaphors and (2) tested these candidates through a multi-method research process to determine their effectiveness in relation to a set of theoretically driven outcomes.

In order to root this process in a deeper understanding of the ways that people think about child abuse and neglect in the UK, we drew on results of a previous study conducted to examine the cultural models [37,38] that members of the British public use to reason about this set of issues [1]. This earlier study employed cultural models theory [39] and methods [40,41] to explore the shared but largely implicit understandings, assumptions, and patterns of reasoning that the British public uses to reason about issues of child maltreatment. Examining these cultural models helps us understand why people struggle to see the causal and preventative role of social determinants in issues of child maltreatment. These cultural models are also helpful in thinking about how metaphors might improve public understanding of the role of context in preventing and addressing child maltreatment. 

Psychological anthropologists and cognitive linguists have studied and documented shared sets of hierarchically “nested” cognitive structures that are used by members of a culture to make sense of experiences and social worlds [38,39]. Cultural models are consistently implied relationships, propositions, and assumptions applied to make sense of information and experiences. Goffman described these models as the shared cultural content that allows us to make sense of an infinite range of incoming information and experiences in order to interact seamlessly with the individuals and situations that we encounter [42]. 

Researchers stress a set of features of cultural models. First, these shared structures of meaning are implicit or, as Quinn (2005) writes, “referentially transparent” [40] (p. 3), meaning that people are not aware of the effect of these mental models in shaping their thinking. Second, these patterns of meaning making are shared [40]. In this way, membership in a cultural group is both defined and facilitated by the possession and use of shared cultural models such that what is taken for granted is taken for granted by all those sharing common models [38]. Put another way, people who share cultural models make a common set of assumptions and, therefore, what is taken for granted is, in fact, tacitly understood by all [42]. Those studying cultural models also note that these models are cued when individuals are engaged in cognitive tasks, like telling stories, explaining an experience, or justifying a decision [40,43].

Below, we review some of the most dominant cultural models that guide and shape the British public’s thinking about early childhood, adversity, and maltreatment. A more comprehensive analysis of these and other patterns of thinking about development and maltreatment can be found elsewhere [1,19]. A major finding of this research is the existence of a set of highly dominant cultural models that lead people to focus on individual causes of and solutions to issues of child maltreatment, while at the same time finding the presence of a set of more backgrounded assumptions and understandings through which people can appreciate more contextual dimensions of child abuse and neglect. Working from this perspective, the idea of reframing the issue of child maltreatment to nurture a social determinants perspective is a task of foregrounding certain existing (although more recessive) ways of thinking about the issue, while backgrounding other ways of thinking that obscure an appreciation of the role and importance of social and contextual determinants on this issue.

To reflect this distinction in public thinking, we organize the presentation of cultural models findings below into those ways of thinking that focus attention narrowly on individual determinants of maltreatment, and those ways of thinking through which people are more able to engage with a *social* determinants perspective on this issue.

## 4. Individualist Cultural Models 

*What Doesn’t Kill You Makes You Stronger*: Participants in our research generally underestimated the negative ways that severe stress can affect development and shape long-term life outcomes. In fact, participants tended to assume that stress in early childhood—even severe stress—strengthens and builds character. Viewing stress as a positive developmental experience through which character and strength develop makes programs that aim to address and reduce sources of stress for young children seem unnecessary, and even counterproductive, limiting public support for strategies designed to address the social determinants of maltreatment.

*Bootstraps*: Participants consistently assumed that the drive and willpower that both the child and his or her parents exert is one of the most important factors shaping a child’s development. From this perspective, there are few obstacles that children and parents cannot overcome through the application of hard work and effort. This perspective focused participants’ attention at the individual level and made it difficult for people to see how external factors and determinants outside of an individual’s control affect both parenting and development. The result is a de-emphasis of the importance of external supports and the potentially damaging effects of severe stress.

*Increase Awareness*: Participants shared an understanding that increasing *awareness* about forms of maltreatment and early adversity is potentially an effective prevention strategy. They reasoned that if more people were aware of the problem of maltreatment, they could better protect their children and perhaps ‘catch’ themselves or others before they committed an act of maltreatment. This is clearly a way of thinking that is connected to the bootstraps model—if only people knew, they would be able to exert will, make better choices, modify behavior, and avoid committing acts of abuse or neglect. This way of thinking obscures an appreciation of social determinants by focusing attention at the individual level.

*Social Class Stereotypes*: Participants had common ways of thinking about “lower class” people. They assumed that lower-class families are less educated and ambitious, less able and willing to provide for their children and, fundamentally, less competent parents. Thus, the public’s stereotypes of lower class families focus on ‘innate’ characteristics of the people themselves (lazy, uneducated, selfish…), rather than on the ways in which social and economic disadvantage might pressure and shape behaviors.

*Selfish Caregivers*: Another highly individualizing way of thinking about child abuse and neglect is the assumption that maltreatment is the result of selfishness and immorality on the part of the caregiver. Participants reasoned that neglect occurs in many cases because the people who are supposed to care for children are more concerned with themselves than for the wellbeing of their children. According to this assumption, people neglect their children because they have not developed the maturity, do not have the moral strength or are not in possession of the character to put their children’s needs ahead of their own.

*Fatalism:* In our earlier research, we found that members of the British public are generally fatalistic about the possibility of reducing early adversity and child maltreatment. This is, in part, because of the factors that people see as causes of adversity and maltreatment—most of which are difficult to see as addressable. After all, how do you reduce someone’s selfishness, eliminate poverty, or reverse the degradation of community and societal values?

## 5. Contextually Sensitive Cultural Models

*The Need for Protection*: Participants shared an assumption that positive development is centrally about protection from physical threats. According to this cultural model, negative development in early childhood results from adults’ failure to adequately protect young children from physical threats in their environment. While this way of thinking helps people recognize that environments can pose threats to child development, it focuses attention quite narrowly on physical threats to the child, excluding many of the social determinants and contextual causes about which experts and advocates would like to communicate.

*Lack of Resources*: Participants assumed that, in many cases, maltreatment—especially neglect—occurs when caregivers lack the financial means to care and provide for children. Neglect, according to this model, occurs when people living in poverty lack the resources to provide adequately for children. This way of thinking about maltreatment allows people to see contextual influences on parenting and development, though it focuses thinking on financial constraints.

*Stress Affects Behavior*: There was also a clear assumption that adults who are ‘stressed out’, overworked, unemployed, or frustrated with life are more likely to take out their frustrations on children either through physical abuse or by becoming distracted from caring for their children, resulting in neglect. Poverty was the most frequently cited source of stress and distraction.

*Government Responsibility*: Through its enforcement agencies (courts, police, social services, and health services), people understand that government has an important role to play in addressing child maltreatment and early adversity. Members of the British public believe strongly that government has a core duty to keep its citizens—and especially its children—safe.

## 6. Metaphor Tasks and Candidates

We used findings from the cultural models analysis to arrive at a set of conceptual “tasks,” or functional outcomes, that we wanted metaphors to be able to address. Metaphor tasks were as follows:

- **Help people think beyond individual-level causes of maltreatment and adversity to see the importance of social causes and potential for social solutions**. Although our earlier research shows that members of the public are somewhat attuned to the role of economic disadvantage in child maltreatment, individual-level explanations are more dominant—easier to “think”, and quick to crowd out more social-level thinking. As a result, it is difficult to see the need for (and potential of) structural, societal-level interventions to address social determinants.
- **Expand people’s understanding of the effects of poverty to include other social determinants**. The public’s social class stereotyping locates maltreatment predominantly in lower/working-class families. This stereotyping blocks thinking about other environmental factors such as work policy, mental health stigma, and substance abuse, all of which can contribute to maltreatment and adversity across social classes.
- **Help people see that addressing the social determinants of child maltreatment and reducing early adverse experiences is possible***.* For a variety of reasons, members of the public are highly skeptical about the possibility of addressing child maltreatment and reducing adversity. This sense of fatalism represents a central communications challenge, as it hampers public support for policies and programs: If a problem cannot be fixed, why spend time and money trying? Effective messaging about the social determinants of child abuse and neglect can show people that change is possible by demonstrating how programs can meaningfully address social factors to reduce maltreatment and prevent adversity in the lives of children.

After specifying the tasks that a metaphor would need to achieve, we generated a list of potential metaphors that seemed, hypothetically, to have the ability to illustrate a social determinants perspective and shift solutions thinking in more contextual and preventative directions. 

**Figure 1:**
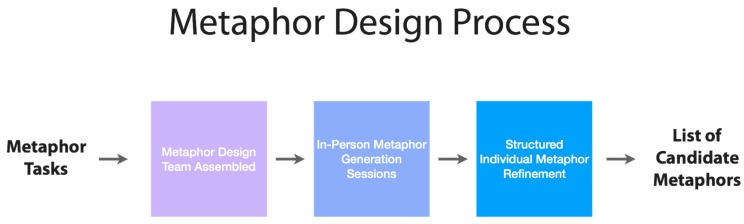
The metaphor design process.

We assembled a 12-person metaphor generation team comprised of applied social scientists; public health scholars specializing in social determinants of health and health communication; researchers and advocates from the field of child abuse and neglect; journalists; communication and public relations professionals; and metaphor scholars. This group participated in three in-person metaphor brainstorming sessions and completed structured individual work in which they generated general metaphor categories (for example, weight, interference, pollution etc.) and specific instantiations of these general categories (overloaded lorry, social static, social smog etc.) that, in theory, seemed promising in addressing the prioritized metaphor tasks. This process lasted for almost two months and produced a set of candidate metaphors that could be tested using social science methods. This metaphor generation process is discussed elsewhere in greater detail [44,45,46] but summarized in Figure 1 below.

The design process resulted in the following eight metaphors, which are presented below.

1. Eroding Support: A person’s ability to care for their children can get worn away over time in the same way that water can wear away the foundation of a building or bridge, making it less stable. Bad social conditions like poverty and violence can erode the mental and emotional foundation that allows people to manage stress and give care and attention to their children.
2. Mental Static: A person’s ability to care for their children depends on being able to tune into children’s needs in the same way that a radio tunes into different signals. Bad social conditions like poverty and violence can create emotional and mental static that block these signals.
3. Social Terrain: When social conditions are smooth, like a well-paved road, people are in a good position to care for and be responsive to their children. The opposite is also true. Bad social conditions, like poverty and violence, put obstacles in the road and create potholes that damage a person’s mental and emotional ability to navigate stress and care for their children.
4. Caring Charge: A person’s ability to care for their children can get drained, just like in a battery. In particular, social conditions like poverty and violence can drain a person’s mental and emotional ability to manage stress and give care and attention to their child.
5. Staying Afloat: Trying to care for children and manage life in bad social conditions is like trying to stay afloat in a boat in rough seas. Parents who are rocked by social conditions like poverty and violence need support to stay afloat and maintain the mental and emotional ability to give care and attention to their children.
6. Overloaded: Trying to care for children in bad social conditions is like trying to drive an overloaded lorry. Social conditions like poverty and violence can overload a person’s mental and emotional capacity to manage stress and give care and attention to their child.
7. Social Smog: A person’s ability to care for their children depends on their social environment in the same way our ability to function depends on the quality of the air that we are surrounded with. Just like air pollution makes it hard to breathe and be active, being surrounded by things like poverty and violence can strain a person’s mental and emotional ability to give care and attention to their child.
8. Signal Interference: People’s ability to care for their children depends on strong signals between them and their children. Just like bad weather interferes with communication between satellites or mobile masts, social storms like poverty and violence can interfere with a person’s mental and emotional ability to tune into their child’s needs and care for them.

Working with this set of tasks and these candidate metaphors, we began an iterative research process to see whether and which of these metaphors could improve understanding of the social determinants of child abuse and neglect. 

## 7. Testing Metaphors 

**Figure 2:**
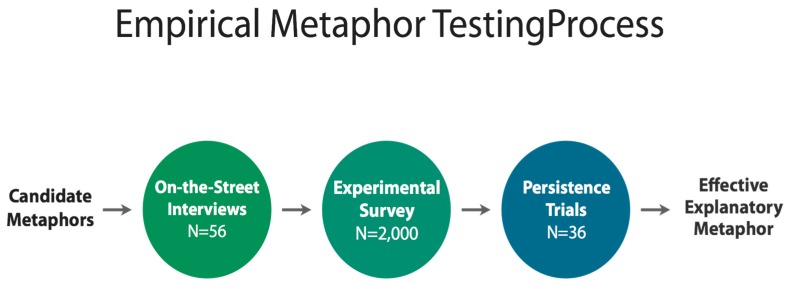
Overview of methods used to test candidate metaphors.

In order to test candidate metaphors for their ability to do the conceptual work that earlier research showed was necessary, we first conducted a set of exploratory on the street interviews with a sample of 56 Britons in two UK locations. Results from these interviews were used to field an experimental survey that tested the three most promising metaphors against a set of relevant dependent variables on a sample of 2000 members of the UK public. Finally, we conducted additional qualitative research, using a method we refer to as Persistence Trials (see Description below) with a sample of 36 members of the UK public. Figure 2 summarizes this research process and below we provide more detailed information about each method. 

### 7.1. On-the-Street Interviews

We used a set of short on-the-street interviews as a way to explore the effects of candidate metaphors. On-the-street interviews were designed as before and after qualitative tests of metaphor effects. Participants were initially asked the following open-ended questions about child maltreatment:

1. What does a child need for healthy, positive development?
2. I’d like to talk a bit about how you think about child neglect. What is child neglect?
  - What are some examples?
  - What would you say causes neglect?
  - What are effects of neglect?
3. What do you think can be done to reduce levels of neglect in the UK?

Each participant was then presented with one of the eight candidate metaphors from the list above. 

Following the presentation of the metaphor, via the text presented above, participants were asked the following questions designed to determine the effect of the metaphor on their thinking and talking about child abuse and neglect:

1. What are your first thoughts about this idea?
2. According to this metaphor, what kinds of things make neglect more likely to happen? (*Restated*: How does it make you think about why neglect happens?)
3. What factors make it more likely that a parent or caregiver will neglect their child?
4. Does the metaphor suggest anything about how we can reduce levels of neglect in the UK?

A total of 56 interviews were conducted in two locations (London and Birmingham). Participants were recruited with an eye for capturing diversity in age, ethnicity, gender, and political affiliation. However, the sample was not, nor was it meant to be, statistically representative. 

All participants were given a verbal and written description of the study before participating and signed written consent forms. All interviews were video- and audio-recorded by a professional videographer. Two researchers independently coded all video data from the interviews using Version 2.5 of Transana (2012) video analysis software (Spurgeon Woods LLC, Madison, WI, USA).

Coding of pre-metaphor data focused on identifying common patters of reasoning, or cultural models, that participants used to make sense of the child abuse and neglect causes, effects, and solutions. In general these responses showed the dominance of the cultural models described in detail above—that people view parental selfishness as the primary cause of child abuse and neglect; that there are strong social class stereotypes about the motivation and caring of particular groups of people (i.e., that lower class people are lazy and have a lower level of caring for their children); that there are robust senses of fatalism about the possibility of meaningfully addressing or preventing child abuse and neglect; and that, where solutions thinking is possible, it focuses squarely at the individual level, centering on parental education and information provision. 

Coding of post metaphor questions focused on whether the metaphors were able to achieve the instrumental goals described above, that is, did post-exposure answers demonstrate thinking that was in line with the metaphor tasks. Coding also was attentive to whether the metaphors were “sticky” in discussion and found their way into answers to post-exposure questions. Examples of generativity and adaptation—whether participants generated their own entailments of the metaphor or used the metaphor to make points that were not included in its initial presentation—were also coded. Finally, gestural uses of the metaphor were coded as evidence of embodiment of the metaphor—a sign of metaphor penetration and usability. Codes were compiled and compared across researchers to arrive at a set of findings as to the general effects of the metaphors tested. The results of this analysis, presented below, were used to select metaphors for the next, quantitative phase of testing. 

Analysis suggested that several metaphors were effective in relation to instrumental outcomes and conceptual metaphor tasks. 

In on-the-street interviews, the Overloaded metaphor was consistently applied to think about a wide range of concepts and relationships that were in line with the metaphor tasks. For example, after being primed with the metaphor, participants were noticeably more focused on contextual factors than in pre-primed discussions. Participants discussed the ways in which a wide range of contextual factors such as employment, childcare, relationships, neighborhood violence, poverty, and racism affect individual behaviors and caregiver decisions and, in turn, increase the likelihood of abuse and neglect. The presence of these contextually focused discussions of social determinants following exposure to the metaphor was a clear pattern in the data, as participants went from highly individualized perspectives in their pre-primed questions, to being much more sensitive and attuned to the role of contextual factors following the presentation of the Overloaded metaphor. The metaphor appeared effective in shifting thinking from highly individualistic causal explanations to an emphasis on external factors and a focus on how context can impinge upon individuals and their decisions and behaviors. 

Analysis also suggested that thinking shifted from highly individualistic solutions to an acknowledgement of the need for measures to address context and provide external supports. 

After hearing the Overloaded metaphor, when asked about what could be done to address issues of child maltreatment participants tended to focus on structural and contextual solutions—for example, improving transportation in underserved neighborhoods, providing more easily accessible, high quality child care, having access to job training programs, and creating more affordable housing options. 

Analysis showed that the Overloaded metaphor was also highly memorable and sticky, with language such as “load”, “loaded”, “overloaded”, “weighted down”, “stacked”, “carrying a load”, “burdened”, “pile”, “piled up” and “piled on” being frequently used by participants in discussion following exposure to the metaphor. The metaphor was also easily incorporated in stories that participants told where characters were described as having to “pull weight” or “deal with heavy loads” and being “weighted down” by contextual and social factors. Participants also used the metaphor in a wide range of non-verbal gestures, for example using their hands to pick up “weight” and move it in and out of space, indicating the difficulty of forward progress of an object when it is weighted down, or noting the differences in steering and navigating in situations of relatively light versus heavy loads. 

Close analysis of these productive effectives showed that they were driven by several features of the metaphor. First, participants used the idea of a lorry as having limited load capacity to communicate the idea that people have limited space/capacity and, therefore, that when presented with challenges there is little space left to deal with other things. This created a zero-sum understanding of attentional resources that allowed participants to see how contextual/social factors affect behavior. The idea that the load of the truck comes from external sources was also an important part of how the metaphor was productively used. When primed with this metaphor participants rarely placed sole responsibility for causes or solutions on an individual person. There was also a kinesthetic principle of weight, effort, and movement that was used productively. People used the idea that the more weight you put on something, the harder it is to move it forward, to discuss the role of contextual factors in influencing individual behavior. Finally, participants used language from a more general journey metaphor and focused on the *external* factors that influence the ability of an agent to move along a trajectory. As one participant said, “You won’t make that journey with all that stuff on your vehicle”.

Staying afloat also performed well in on-the-street interviews. Participants consistently applied the metaphor in answering post-prime questions, and participants exposed to the metaphor consistently generated vivid descriptions of the turmoil that people experience when faced with various aspects of bad weather on the water. Participants connected these descriptions of challenges in the target domains, and focused on the ways in which financial problems, mental health issues, education, and employment issues could affect individual behaviors and outcomes. As the following quotes show, participants used the staying afloat language to talk about social and contextual factors that influence child maltreatment:

“The boat would be the family and the life, the rough seas are things battering them, they don’t know how to redo the woodwork, keep the water out […] problems are multifaceted, complex. They’re not being given the resources to help them keep that boat intact and safe from the storm.”

“Because the seas are so rough, by the time you’ve got to the top of the wave and you hope it’s going to calm down, what’s basically happened is you’ve got another issue, initially it was rough weather, now we’ve got a storm coming and lightening coming. So you weather the storm and all of a sudden you find out there’s no wind and you can’t sail, or the engines have gone. You’re always firefighting. So you don’t have the time you need to nurture your children properly. Overcome that by taking the boat out of the rough sea, put it in a calm harbor with nice high walls so the sea can’t hit you.”

In terms of solutions, participants exposed to the metaphor talked about the need for institutions and organizations that could respond when someone, in the words of one participant, “sends an SOS”.

Finally, the Eroding Support metaphor was promising during these exploratory interviews. Participants exposed to this metaphor focused on the importance of social and systemic factors in understanding the prevalence of abuse and neglect, and on the ways in which these factors undermine parental capacity. The metaphor also was used to talk about the importance of public resources in addressing these social factors. 

The language and concepts of “erosion”, “wearing away”, “getting worn down” and undermining “strong foundations” were persistently used in discussions following exposure to the metaphor. The temporal entailment of the metaphor—that negative social conditions act on people over time and gradually “wear away” their capacity—was frequently evoked to talk about the connections between social determinants and child maltreatment. Participants exposed to the Erosion metaphor focused on how negative social factors make neglect more likely in a society by undermining caregiver capacity. 

Several of the metaphors piloted in on-the-street interviews showed more mixed results. For example, social static was sticky as a term, but was difficult for participants to apply in thinking about child abuse. Other metaphors led in unproductive directions. For example, participants exposed to the Social Terrain metaphor focused on the “traveler” and the need for the individual in question to “overcome obstacles” by making good decisions and trying harder. Other metaphors, such as Social Smog, were poorly understood and had little effect in relation to the metaphor tasks. These metaphors were not brought forward into the next round of testing. 

### 7.2. Quantitative Framing Experiment 

The three most promising metaphors described above (Overloaded, Afloat, and Erosion) were tested in an online experiment [47,48,49] conducted in November 2014. The goal of the experiment was to gather statistically meaningful data on metaphor effectiveness, providing an additional basis for selecting a metaphor that was successful in relation to metaphor tasks. 

The survey was conducted with 4550 members of the UK public selected to represent census figures with respect to gender, age, race/ethnicity, education level, income level, residential location (urban, suburban, rural), and political party identification. The experiment included manipulations and treatments not reported on here; of the 4550 sample, 500 respondents were assigned to each of the three metaphor treatments described below and 500 were included in the control condition for a total of 2000 respondents who participated in the metaphor component of the survey experiment. 

Survey Sampling International (SSI) was used to provide the sample and administer the survey. Respondents were recruited via multiple channels, including through loyalty programs, online advertising, and newspaper and magazine postings. SSI incentivizes panelists through a system in which respondents receive free or discounted internet services, points that can be redeemed for gift cards or cash payments similar to those offered in Amazon’s Mechanical Turk service. 

As with all opt-in panels, it is not possible to compute response rates, although studies have shown that participation rates are broadly comparable for probability and non-probability based samples [50]. In addition, non-probability samples, such as the one used here, are commonly used in survey experiments, where the ability to generalize to a population is less important than the ability to detect the effects of an experimental treatment [51]. Finally, to decrease sample bias, data were weighted in analysis to assure national representativeness in the demographic categories listed above [52]. 

Following random assignment to one of three experimental conditions or a control condition (in which participants received no stimulus), each participant was asked to read an iteration of one of the candidate metaphors, or, in the case of the control condition, was told that they would be answering questions about child abuse and neglect. Each metaphor treatment was designed to be roughly equal in length and parallel in structure to ensure that differences in effect were attributable to core features of the metaphors tested. Additional questions were included in the experiment to assure that the metaphors had in fact been understood and were being used in thinking. 

Metaphor treatments were as follows: 

**Overloaded:** Trying to care for children in bad social conditions is like trying to drive an overloaded lorry. The weight of experiences like poverty and violence overloads a person’s mental and emotional capacity to manage stress and give care and attention to their children. Over time, this heavy load puts a strain on people, and leads to things like mental health issues and substance abuse problems that weaken people’s ability to care for children. However, a lorry can only bear so much weight before it stops moving forward and when an especially large burden—like the loss of a job—is loaded onto an already overloaded lorry, it causes a breakdown of care. However, just like we can unload an overloaded lorry by bringing in other lorries or moving some cargo by train instead, we can provide social supports that offload sources of stress from overloaded parents and improve their capacity to care for their children. Social supports keep families’ lorries moving forward in bad conditions.

**Staying Afloat:** Trying to care for children in bad social conditions is like trying to stay afloat on rough seas. Imagine a boat, with parents and their children on it. When rough waters rock the boat—experiences like poverty and violence—parents need support to stay afloat and maintain the ability to care for their children. Without support, parents have to use their energy to constantly bail water and watch for the next storm. The stress of those storms leads to mental health issues and substance abuse problems that make it even harder to stay afloat and care for children. And when an especially large wave hits—like losing a job—it completely tips the boat over. However, just like we can build harbors where boats can take shelter, we can provide social supports that shelter people during stormy and stressful times. We can give them a chance to repair their boats and regain their capacity to care for their children. Social supports keep these boats afloat in bad conditions.

**Eroding Support:** People’s ability to care for their children can get worn away over time in the same way that water wears away the foundation of a building or bridge, making it less stable. Experiences like poverty and violence erode the mental and emotional foundation that allows people to manage stress and give care and attention to their children. This gradual wear causes mental health issues and substance abuse problems, which weaken people’s ability to care for their children. And when particularly heavy rain comes—like the loss of a job—it further erodes people’s ability to care. However, just like we can bring in engineers and builders to repair the damage caused by erosion and prevent it from happening again, we can provide social supports that strengthen and repair people’s ability to deal with stress and care for their children. Social supports keep parents’ foundations solid in bad conditions.

All participants then answered a set of the same 17 multiple choice questions (randomized). Questions were designed to measure a set of outcomes that were derived from our metaphor tasks. The following are sample questions (the full set of questions is presented in Appendix A): 

Which of the following makes child neglect more likely to happen?

- When parents regularly confront stressful and difficult situations in their lives and communities.
- When parents are selfish and aren’t willing to sacrifice their own wants for the sake of their children’s needs.
- Neglect is part of human nature—there is not anything that makes it more likely to happen.

Which of the following is most likely to prevent neglect from occurring?

- Social supports that prevent family isolation.
- Increased police presence in communities.
- Little can be done to prevent child neglect.

Answers that were consistent with the metaphor tasks (described above) were each awarded one point. Points were summed and averaged for each metaphor and the control condition to create an aggregate measure of metaphor effectiveness. The graph below provides these average scores as well as the percentage change in desired responses for each treatment when compared to the control.

Among the three metaphors tested, the quantitative experiment provided evidence for the effectiveness of the overloaded metaphor. Using *T* tests, differences in effects between the overloaded metaphor and the other two metaphors were statistically significant at *p* < 0.01 Differences between each of the three metaphors and the control condition were also statistically significant at *p* < 0.01. Differences between the Overloaded metaphor and the two other metaphors were significant at a lower level of statistical significance (*p* < 10). 

### 7.3. Persistence Trials

**Figure 3:**
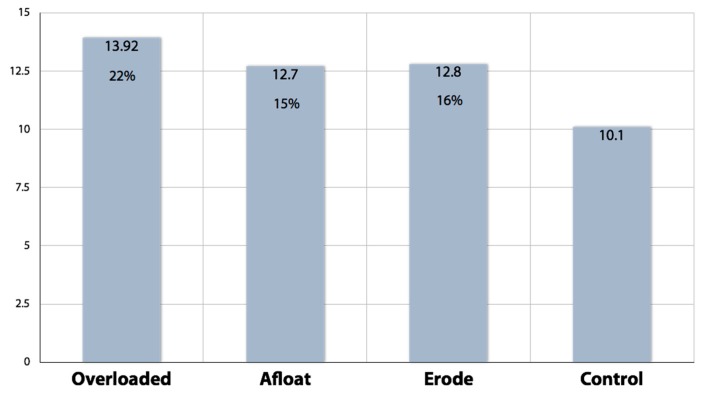
Results from the quantitative framing experiment.

Results from the quantitative metaphor experiment are summarized in Figure 3 and were used to select the most promising metaphor—the overloaded metaphor—which was refined using a final qualitative method that we refer to as “persistence trials” [26,53].

Persistence trials are designed to answer two questions: (1) can and do participants transmit the metaphor to other participants with fidelity? and (2) how do participants transmit the metaphor? We used the method here to examine how well the overloaded metaphor holds up when being ‘passed’ between individuals, and how participants use and incorporate the metaphor in discourse. 

Six persistence trials were conducted in January 2015 between two locations (London and Manchester) with a total of 36 participants, selected by a professional marketing firm to include variation on key demographic variables: age, gender, education, income, residential location, and political identification. 

A persistence trial begins with two participants who are presented with a metaphor. The following metaphor was presented to participants:

The weight of life’s challenges can overload a person’s mental and emotional capacity to manage stress and give care and attention to their children. Over time, this heavy load puts a strain on people, and can lead to things like mental health issues and substance abuse problems that weaken people’s ability to care for children. Just like a lorry can only bear so much weight before it stops moving forward, when an especially large burden is loaded on a person who is already over-loaded, it can cause a breakdown in care. This doesn’t mean that bad social conditions always lead to neglect, but that these kinds of conditions make it more likely that care will break down. However, just like we can unload an overloaded lorry by bringing in other lorries or moving weight in other ways, we can provide social supports that offload sources of stress from overloaded parents and improve their capacity to care for their children. This is how social supports can help keep families moving forward, even in bad conditions.

Participants were then asked a series of open-ended questions designed to gauge their understanding of the metaphor and their ability to apply the metaphor in discussing causes and solutions to child maltreatment. Questions were also designed to locate any terms or ideas in the execution of the metaphor that participants had difficulty with or explicitly recognized as problematic.

Questions included the following:

1. What is the main idea that you got from this?
2. How would you describe what child abuse and neglect are to someone who is unfamiliar with this term?
3. Tell me about what you think causes child abuse and neglect—why do these things happen?
4. Why do some parents abuse or neglect their children?
5. What do you think could be done to address the problem of child abuse and neglect?

After discussion between two initial participants (Generation 1) and the researcher, Generation 1 was informed that they would be teaching the metaphor to another pair of participants (Generation 2). Generation 1 was then given five min to design a way of using the metaphor to present about child maltreatment, after which they had five min to present to Generation 2. Generation 2 then had five to 10 min to ask Generation 1 questions about the presentation. 

Generation 1 then left the room and the interviewer asked Generation 2 an additional set of questions designed to elicit their understanding of the issue and the metaphor. This questioning lasted for approximately 10 min, at which point Generation 2 was informed that they would be using the metaphor to present on the issue to two new participants (Generation 3). Generation 2 had five min to plan their presentation, after which Generation 3 entered the room and the two groups went through the same steps and questions as described above.

A persistence trial ended when Generation 1 returned to the room. Generation 3 presented to Generation 1, after which there was a debrief with Generation 1 and Generation 3 on the direction the metaphor had taken over the course of the session.

All video data from the sessions were coded and analyzed independently by two researchers. Coding focused on identifying examples of metaphor application and lack thereof; communicability and failures in transmission; and specific language of usage. Coded sections of the sessions were subjected to a deeper qualitative analysis to answer the following questions:

1. Were participants able to apply the metaphor; and, more specifically, what were the ways in which they applied it?
2. Was the metaphor communicable? Were each Generation’s presentations of the metaphor faithful to the initial model presented by the researcher? How did the groups’ presentation of the metaphor differ from the researcher’s initial presentation?
3. What specific language did the groups use in discussing the metaphor? Was there language that participants used that was not included in the original execution of the metaphor?

Participants in the trials consistently used the overloaded metaphor to discuss the role of social conditions in child abuse and neglect. The metaphor was used in discussions about how social conditions such as financial insecurity, access to quality public services, and community violence contribute to abuse and neglect. The metaphor was also used in talking about issues beyond child maltreatment, including addiction, mental health, and physical health and helped participants see the more general connections between social determinants and health and wellbeing.

The idea that ‘weight can overload a lorry’, was used by participants to discuss the role that social conditions can play in child maltreatment. The metaphor was also applied in discussions about how experiences of adversity in early childhood can affect development and shape long-term capacities—for example, there were frequent discussions about how bearing a lot of weight from an early age might compromise systems and capacities (e.g., stressing the engine or axels) and make a person less able to respond productively to challenges and burdens as a young parent. 

Discussion in persistence trials also focused on solutions—in particular, the ways that external supports can ‘offload’ sources of stress to improve functioning. For example, participants described how social workers can put up ‘signposts’ that help lorries figure out where to go for support and nurses can identify the weights that are heaviest and look for ways to “pick them off”. The metaphor was also used to discuss how abuse and neglect can, through the right interventions, be prevented. Participants used the metaphor to discuss how social systems and services can act as a form of ‘maintenance’ to increase resilience and reduce the likelihood of a person becoming overloaded in the first place. 

Analysis showed a set of particularly sticky terms associated with the metaphor including “load”, “loaded”, “overloaded”, “weighted down”, “stacked”, “carrying a load”, “burdened”, “pile” and “pile on”. Persistence trials showed that participants could easily take up and use this language in having productive conversations focused on social determinants of child abuse and adversity. 

**Figure 4:**
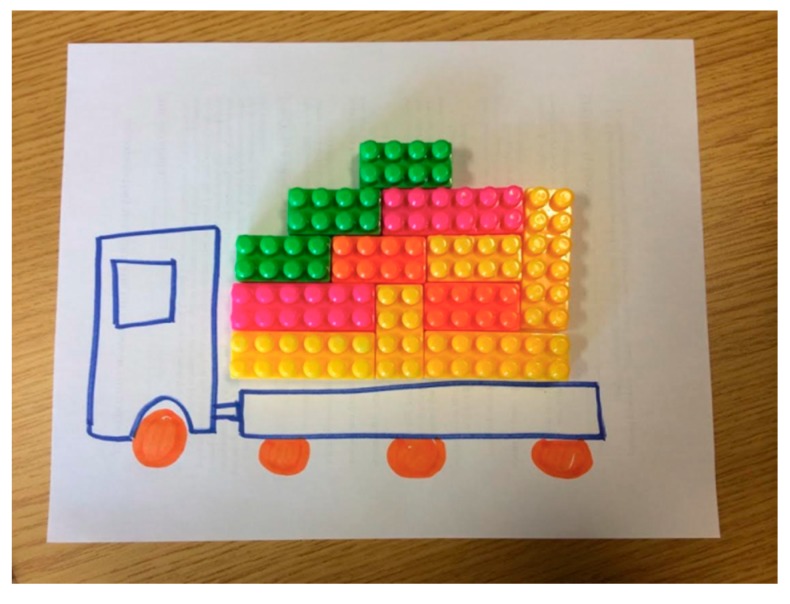
Image of participant depiction of the metaphor in a persistence trial.

Finally, the Overloaded metaphor was highly visual, and lent itself to illustration and manipulation of physical materials. For example, one pair of participants drew an outline of a lorry see Figure 4) and used different colored Legos to illustrate how weight would affect forward progress and to discuss strategies that would reduce the likelihood of abuse and neglect by offloading different weights through social programs and policies. 

Following analysis of data from persistence trials, we refined the metaphor and constructed the following iteration to guide communicators in using the idea:

Overloaded: When a lorry carries too much weight, it can be overloaded to the point of breaking down. And when parents are burdened with stresses like poverty or lack of support, the weight of these problems can overload their mental and emotional capacity to take care of their children’s basic needs. Over time, carrying and managing heavy burdens puts a strain on people, and can weaken their ability to care for children. And when an especially large burden is loaded onto a person who is already overloaded, it can cause a breakdown in care. However, just like we can unload an overloaded lorry by sharing the load with other lorries or offloading cargo in other ways, we can provide social supports that offload sources of stress from overloaded parents and improve their capacity to care for their children.

## 8. Discussion 

This research suggests that metaphors can be effective in communicating a social determinants perspective through which people can think about the causes and consider the potential solutions to issues of child maltreatment. These findings are evident through comparisons of pre- and post-exposure discussions in exploratory on-the-street interviews; by comparing the control and treatment conditions in the experimental survey; and by comparing the results of previous qualitative in-depth interviewing [1] with the results from persistence trials.

While this research suggests that metaphors are helpful devices in communicating a social determinants perspective, there are a number of ways in which our research is limited in its ability to address on this question. First, and most importantly, the research is limited by its inclusion of a null condition as the sole control in the experiment. This allows us to say that metaphors appear more effective than merely cuing the domain of child abuse and neglect, but does not allow us to comment on whether or the degree to which metaphors are more effective than non-metaphorical explanations of social determinants. Future research could replicate the metaphor experiment and run multiple controls to more fully answer the question of whether metaphors are more effective than other means of explanation in improving understanding of the social determinants of child abuse and neglect. Our ability to comment on the effectiveness of metaphor in relation to other framing strategies is further limited by the fact that we did not test other, non-metaphorical frames, such as values, messengers, statistics, narratives, or solutions frames. Future research should explore the ability of other frame elements to affect thinking on the social determinants of abuse and neglect. 

Another limitation is that our work examined patterns of talking, thinking, and answering closed ended questions, but did not attempt to measure the effects of metaphors on other important outcomes, such as efficacy, motivation to engage, or a range of behavioral outcomes. Research that explored the effect of metaphors on different types of outcomes would help advance our knowledge of the effects of metaphor on health and social issue communication. 

In addition, findings from our research suggest that there are differential effects between metaphors in relation in their ability to increase the availability of a social determinants perspective. We find that one metaphor, at least among the eight tested here, is particularly effective in expanding people’s thinking beyond individual level causes and solutions to include an acknowledgement of the role that social and contextual factors play in child abuse and neglect. This finding emerges from comparing post-exposure answers across different metaphors and comparing the effects of different metaphor treatments in the quantitative experiment. However, our ability to make claims about differential effects across metaphors is also limited by aspects of our design and analysis—limitations that could be addressed in subsequent research employing different designs. 

First, while we were careful to titrate metaphor presentations in qualitative and quantitative work to assure that the metaphor in question was in fact the operative independent variable, it remains possible that, even across multiple methods, there was another component of the treatment that was driving the effects that we observed. Future research could use different methods to provide additional evidence that the differential effects observed across metaphors are, in fact, due the metaphors themselves. 

There is also an important set of application questions that are at least partially unanswered by the current research: How can communicators best use the overloaded metaphor? What entailments of the metaphor are most effective in producing effects on social determinants thinking? Is there a way of improving on the iterations tested to generate more effective linguistic applications of the metaphor? What about non-linguistic cues—can they be used to activate the metaphor? How? In short, there are numerous unanswered questions that are of applied importance to those who would use this metaphor as a communications tool. Future research could test the effects of ways of iterating the metaphor as a way to answer some of these questions. 

There are also important, but unanswered questions about the generalizability of the metaphor beyond child abuse and neglect. Can the overloaded metaphor be used to make more general points about social determinants, or to effectively deliver specific messages on other health issues? We would encourage other researchers to explore these questions. 

A final and significant limitation of our work is that we only tested eight metaphors through the course of this research. It may very well be that there is another metaphor that would be more effective than was the overloaded metaphor in relation to our instrumental tasks. Future research could test a more comprehensive set of metaphors to potentially find something superior to the overloaded idea. 

## 9. Conclusions 

Our question in conducting this research was: can metaphor be used to improve understanding of the social determinants of child maltreatment and increase support for preventative policies and practice? We found that metaphor in general, and one metaphor in particular had positive effects in expanding public thinking about the causes of and solutions to issues of child abuse and neglect. The overloaded metaphor broadened the set of factors that people could see as causes of abuse and neglect and shifted their focus from individual level solutions to a more comprehensive set of actions that could be taken to address this issue. In particular the metaphor increased people’s openness to see the importance of contextual and social level changes as ways to address and prevent child maltreatment. The metaphor, in doing this deep work, seems promising as a way to increase people’s recognition of the role of social determinants in other health and social issues. 

Better understanding of the social determinants of abuse and neglect is a vital precursor to support for the policies and programs that can address and prevent these issues. We argue, therefore, that communications and framing are key dimensions of this work. 

This kind of communications work has been a significant activity for the NSPCC. The organization’s communications work has gone beyond the standard fare of awareness-raising and fundraising. Instead it has attempted to use metaphors like the one described here to offer explanations about the causes of child abuse and neglect, and to describe solutions. The NSPCC has developed specific assets which communicate this and other tested metaphors, values and principles, for example, an animation which deploys these frames to clearly and effectively communicate about why child abuse and neglect happens, the consequences of these issues for wider society, and what can be done to prevent and remediate the effects of child maltreatment. 

The NSPCC is evaluating the impact of using these new evidence-based communications tools, by tracking public beliefs and attitudes in ways that offer at least correlational evidence that significant communications efforts and messaging campaigns are related to changes in understanding, attitudes and solutions support, for example. Evidence-informed communications are playing an increasing role in strengthening support for social change on child abuse and neglect throughout the UK, and improving public understanding of the social determinants of these issues is a major part of this work. 

As more and more organizations in the UK seek to improve public understanding of the social drivers of early adversity and maltreatment, our research highlights a number of central communications challenges that must be addressed to build this public understanding. These include: (1) explaining how social conditions can lead to maltreatment and adversity; (2) helping members of the public understand how adversity affects child development; (3) showing social conditions as dynamic and tractable, rather than intransigent unchangeable; and (4) demonstrating the role of social supports in addressing and preventing child abuse and neglect. 

Through our work we find one framing tool, a metaphor, that effectively meets these communications challenges, and can help those working in public health and human services to communicate about the social drivers of maltreatment and childhood adversity. 

## Appendix A

Questions from the Quantitative Experiment (answer order was randomized in the experiment, however, in the questions below, answer “a” is desired answer in each item).

1. Which of the following best describes child neglect?
  - Consistently failing to provide for a child’s basic needs.
  - Leaving a child with a nanny for extended periods.
  - Being late to pick up a child from school or daycare.
2. Which of the following do you most agree with?
  - Child abuse and neglect is more likely to happen when a parent’s capacity and ability to care is weakened.
  - Child neglect happens when a parent is selfish and chooses not to care for their child.
  - Child neglect happens when a child is consistently disobedient to their parents.
3. Which of the following do you most agree with?
  - Child neglect is the lack of care over time.
  - Child neglect is normally an unavoidable part of life.
  - Child neglect is something that children naturally get over.
4. Which of the following would you say is most true?
  - At its core, neglect is about how well a child is cared for.
  - At its core, neglect is about how well a child is disciplined.
  - At its core, neglect is the result of selfish parents.
5. Which of the following factors contributes most to abuse and child neglect in the UK?
  - Negative social and economic conditions.
  - The selfishness or laziness of parents.
  - A growing number of people from other countries living in the UK.
6. Which of the following makes child abuse and neglect more likely to happen?
  - When parents regularly confront stressful and difficult situations in their lives and communities.
  - When parents are selfish and aren’t willing to sacrifice their own wants for the sake of their children’s needs.
  - Abuse and neglect are part of human nature—there is not anything that makes it more likely to happen.
7. Which of the following would you say is the strongest cause of child abuse and neglect?
  - Life circumstances that create chronic stress for parents.
  - Parents who have jobs that keep them at work for long hours.
  - We do not know much about what causes child neglect.
8. Which of the following do you think is a significant cause of child abuse and neglect?
  - A stressful environment—it disrupts parents’ ability to take care of their children.
  - Parent’s personality—some people are simply more likely to neglect their children than others.
  - Wealth—the drive to make money distracts people from caring for children.
9. Which of the following do you most agree with?
  - When parents are overwhelmed, they are more likely to mistreat their children–even though they don’t want or intend to.
  - Even when parents are overwhelmed, they still have the ability to choose to care for their children.
  - There isn’t really anything that makes a parent more likely to mistreat a child.
10. Which of the following do you most agree with?
  - Long-term hardship can weaken a person’s ability to care effectively for their children.
  - Long-term hardship strengthens a person’s ability to care effectively for their children.
  - Long-term hardship doesn’t have an effect on a person’s ability to care effectively for their children.
11. Which of the following statements do you most agree with?
  - Parents dealing with mental health issues often need extra help to develop their ability to care for their children.
  - Parents dealing with mental health issues need to take responsibility for their problems and take control of their lives.
  - Parents dealing with mental health issues should choose to have other family members take care of their children.
12. Which of the following would be the best way to address child abuse and neglect?
  - Improve the quality of and access to mental health services for parents.
  - Punish parents who neglect their children.
  - There is no good solution to this issue—abuse and neglect are just part of human nature.
13. Which of the following is the best course of action when a case of child abuse or neglect is identified?
  - Provide support for parents to help them better care for their children.
  - Immediately and permanently remove the children from the home.
  - It is important not to interfere in private family matters, so avoid doing anything until things get really bad.
14. Which of the following actions should be taken when there is a suspected case of child abuse or neglect?
  - Trained professionals should visit the home to assess the situation.
  - The police should be called to come remove the child from the home.
  - Nothing should be done until it is proven that the child is being abused or neglected.
15. Which of the following would be most likely to prevent child neglect?
  - Improve quality and access to drug treatment to parents when they need it most.
  - Little can be done, because it really just depends on what sort of people the parents are.
  - Figure out who is likely to be a bad parent and encourage them not to have children.
16. Which of the following is most likely to prevent abuse and neglect from occurring?
  - Social supports that prevent family isolation.
  - Increased police presence in communities.
  - Little can be done to prevent child neglect.
17. Which of the following would be the best action for communities and government to take in the effort to end child abuse and neglect in the UK?
  - Strengthen mental health and substance abuse services.
  - Strengthen people’s awareness of how common child abuse is.
  - Strengthen punishments for people who neglect their children.
